# High Antibody Titer against Apical Membrane Antigen-1 Is Required to Protect against Malaria in the Aotus Model

**DOI:** 10.1371/journal.pone.0008138

**Published:** 2009-12-03

**Authors:** Sheetij Dutta, JoAnn S. Sullivan, Katharine K. Grady, J. David Haynes, Jack Komisar, Adrian H. Batchelor, Lorraine Soisson, Carter L. Diggs, D. Gray Heppner, David E. Lanar, William E. Collins, John W. Barnwell

**Affiliations:** 1 Department of Epitope Mapping, Walter Reed Army Institute of Research, Silver Spring, Maryland, United States of America; 2 Malaria Branch, Division of Parasitic Diseases, Centers for Disease Control and Prevention, Atlanta, Georgia, United States of America; 3 Malaria Vaccine Development Program, United States Agency for International Development, Washington, D. C., United States of America; 4 Division of Malaria Vaccine Development, Walter Reed Army Institute of Research, Silver Spring, Maryland, United States of America; University of California Los Angeles, United States of America

## Abstract

A *Plasmodium falciparum* 3D7 strain Apical Membrane Antigen-1 (AMA1) vaccine, formulated with AS02_A_ adjuvant, slowed parasite growth in a recent Phase 1/2a trial, however sterile protection was not observed. We tested this AS02_A_, and a Montanide ISA720 (ISA) formulation of 3D7 AMA1 in Aotus monkeys. The 3D7 parasite does not invade Aotus erythrocytes, hence two heterologous strains, FCH/4 and FVO, were used for challenge, FCH/4 AMA1 being more homologous to 3D7 than FVO AMA1. Following three vaccinations, the monkeys were challenged with 50,000 FCH/4 or 10,000 FVO parasites. Three of the six animals in the AMA+ISA group were protected against FCH/4 challenge. One monkey did not become parasitemic, another showed only a short period of low level parasitemia that self-cured, and a third animal showed a delay before exhibiting its parasitemic phase. This is the first protection shown in primates with a recombinant *P. falciparum* AMA1 without formulation in Freund's complete adjuvant. No animals in the AMA+AS02_A_ group were protected, but this group exhibited a trend towards reduced growth rate. A second group of monkeys vaccinated with AMA+ISA vaccine was not protected against FVO challenge, suggesting strain-specificity of AMA1-based protection. Protection against FCH/4 strain correlated with the quantity of induced antibodies, as the protected animals were the only ones to have in vitro parasite growth inhibitory activity of >70% at 1∶10 serum dilution; immuno-fluorescence titers >8,000; ELISA titers against full-length AMA1 >300,000 and ELISA titer against AMA1 domains1+2 >100,000. A negative correlation between log ELISA titer and day 11 cumulative parasitemia (Spearman rank r = −0.780, p value = 0.0001), further confirmed the relationship between antibody titer and protection. High titers of cross-strain inhibitory antibodies against AMA1 are therefore critical to confer solid protection, and the Aotus model can be used to down-select future AMA1 formulations, prior to advanced human trials.

## Introduction

A vaccine based on a recombinant circumsporozoite protein, RTS,S was shown in a recent phase 2 clinical trial in 5–17 month old children to have 53% vaccine efficacy against *P. falciparum* malaria clinical episodes. [1]. One strategy to build on the success of this vaccine is to combine it with other *Plasmodium* antigens [2]. *P. falciparum* Apical Membrane Antigen-1 (AMA1) is one such promising malaria vaccine candidate [3]. AMA1 is located within the micronemes of *Plasmodium* merozoites present within blood stage schizonts. At the time of schizont rupture the AMA1 protein gets translocated to the merozoite surface [4], where it plays a vital role in the erythrocyte invasion process [5]. Antibodies against AMA1 are potent inhibitors of merozoite invasion [6]. AMA1 is also present on the surface of sporozoites and AMA1 antibodies inhibit sporozoite invasion into hepatocytes [7].

In the first non human primate vaccine trial reported for AMA1, three rhesus monkeys received 2 doses of an affinity purified native *P. knowlesi* AMA1 protein vaccine adjuvanted with saponin [8]. Following challenge all three control monkeys required radical drug cure due to acute parasitemia. Two of 3 AMA1 immunized monkeys had parasitemia profiles similar to that of the controls, but one monkey showed a brief delay in patency followed by a self-limiting infection. Upon re-challenge, all 3 immunized animals showed near sterile protection. In a repeat experiment, 3 monkeys received 3 doses of the AMA1 vaccine. As in the first experiment, only 1 out of the 3 monkeys showed a degree of protection. Hence, vaccine efficacy as measured by reduced parasite burden was 33% and as measured by sterile protection was 0%. Notably serum from the protected monkeys had the highest pre-challenge parasite invasion inhibitory activity against *P. knowlesi* merozoites [8].

A subsequent immunization trial used recombinant *P. fragile* AMA1 in squirrel monkeys [9]. Five *Saimiri* (squirrel) monkeys received 3 doses of a baculovirus-produced recombinant *P. fragile* AMA1 protein vaccine adjuvanted with Montanide ISA720. Following challenge with *P. fragile* blood stage parasites, all 4 control monkeys required drug treatment for high parasitemia. Four out of the 5 AMA1 vaccinated animals showed suppressed parasitemia profiles compared to the controls. The efficacy of this AMA1 vaccine as measured by reduced parasite burden was 80% and as measured by sterile protection was 0%. Serum from the one non-protected animal had the lowest IFA titer while the two best protected animals had the highest titer in the trial. [9].

Another trial used *P. cynomolgi* as a challenge parasite in macaque monkeys [10]. Five rhesus monkeys were vaccinated with 3 doses of yeast recombinant *P. vivax* AMA1 adjuvanted with SBAS2 (AS02_A_). These monkeys were then challenged with the heterologous, yet closely related simian malaria parasite *P. cynomolgi*. However, the course of infection of the AMA1 immunized animals was not different from the control group [10].

Recombinant AMA1 of the *P. chabaudi* rodent malaria parasite also protected vaccinated mice against lethal challenge with a homologous strain of *P. chabaudi*, but no protection was observed against a heterologous strain [11]. As above in primate models, the vaccine efficacy in the rodent models correlated with antibody titers [12] and protection was also conferred upon passive transfer of IgG. Antibodies against conformational epitopes were critical for this protective response [11]. Thus, AMA1 based protection appears to correlate best with antibody levels against conformational epitopes that are strain specific.

More recently and of greater relevance, *Aotus* monkeys were vaccinated with a recombinant *P. falciparum* FVO strain AMA1 protein adjuvanted with Freund's complete/incomplete adjuvant [13]. Following challenge with the homologous FVO strain of *P. falciparum*, 9 of 9 control animals developed high parasitemia and required drug treatment within 15 days of challenge. Of the 6 AMA1 vaccinated animals, 3 showed strong indications of protection (2 were subpatent and 1 had delayed patency). The remaining 3 monkeys developed rapidly rising parasitemia and required treatment for either high parasitemia or anemia. Efficacy as measured by reduced parasite burden was 3/6 (50%) and as measured by sterile protection was 2/6 (33%) [13]. Although, the two monkeys that showed sterile immunity had high ELISA titers, a third monkey with comparable ELISA titer was not protected in the study.

Based on the promising preclinical evidence, Walter Reed Army Institute of Research has developed a 3D7 AMA1 vaccine [2]. This vaccine is under advanced stages of clinical evaluation for efficacy in humans [14], [15]. A recent study showed that while the AS01_B_ and AS02_A_ adjuvanted vaccine did not induce protection in humans upon challenge, both had a slight effect on the growth rate of the homologous strain parasite in the blood as observed by quantitative PCR [16]. We present here results from an immunogenicity and efficacy trial with the 3D7 AMA1 vaccine in an *Aotus nancymaae* monkey model, conducted at the Centers for Disease Control (Atlanta GA). Two human-use adjuvants, Montanide ISA720 and AS02_A_ were used in the trial. Since the 3D7 strain of *P. falciparum* does not invade Aotus RBC's [17], two heterologous parasite strains, FCH/4 and FVO, were used for the challenge [18], [19], the former strain being much more homologous to 3D7 than the latter strain.

## Materials and Methods

### Ethics Statement

This study was approved by the CDC Institutional Animal Care and Use Committee. Animals were housed at a CDC primate facility fully accredited by the Association for Assessment and Accreditation of Laboratory Animal Care International (AAALAC). Aotus monkeys were pair housed, under space recommendations for lab animals set forth by the Care and Use of Laboratory Animals, NIH. Monkeys were fed a diet that was shown to provide adequate nutrition and calories in captive *Aotus* used in malaria research. Animals were weighed at weekly intervals and treated for veterinary problems as they arose by an attending veterinarian. All observations were recorded and entered into a computer database. Blood collections of no more than 5% of a monkey's total blood volume were made by venipuncture and taken biweekly. Persons responsible for handling the monkeys and reading smears for parasitemia did not know the group to which the animal was assigned. All vaccine formulations were free of endotoxin and only human-use adjuvants were used in the study. Monkeys that developed high-density parasitemia (>200,000 parasites/µL of blood) or anemia (hematocrit below 20%) were cured with drugs and treated by iron supplementation and transfusion of whole blood.

### Animals and Parasites

Following an initial quarantine period monkeys with intact spleens and no history of *Plasmodium* infection were selected for the study. Monkeys were in good health, free of tuberculosis and weighed between 700–1000 g at the start of the study. Thirty animals were stratified into groups of six animals according to weight and sex. Groups were then assigned randomly to the vaccine and control groups. Challenge infection was conducted using two *Aotus* monkey adapted *P. falciparum* strains. The FCH/4 strain was isolated in the Philippines and produces a moderately virulent infection in spleen intact *Aotus nancymaae* monkeys with ∼50% of monkeys requiring antimalarial treatment following an acute rapidly rising parasitemia. The other ∼50% of animals resolve the parasitemia before reaching 200,000 parasites per µL of blood [18]. The Vietnam Oak Knoll (FVO) strain was isolated from a soldier returning from Vietnam and produces a highly virulent infection with >90% of spleen intact *Aotus nancymaae* requiring antimalarial treatment [19].

### Vaccine

**Figure 1:**
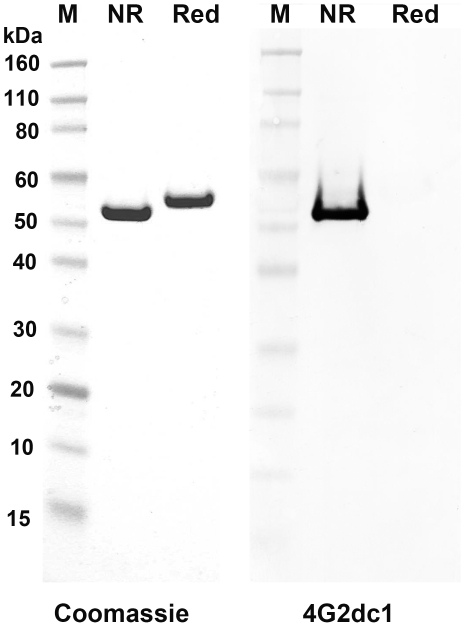
Recombinant 3D7 AMA1 vaccine was pure and folded correctly. Left panel shows a coomassie blue stained SDS-PAGE analysis of the 3D7 AMA1 vaccine under non-reducing (NR) and reducing conditions (Red). Right panel shows positive reactivity of only the non-reduced 3D7 AMA1 protein with a conformational monoclonal antibody 4G2dc1.

The recombinant 3D7 AMA1 vaccine comprising amino acid number 83–531 was expressed in *E. coli* [20]. The 3D7 AMA1 protein used in this trial was produced under GMP conditions, at the WRAIR bioproduction facility. The process used for manufacturing the antigen was essentially as described in Dutta *et al.* 2001 [20], with one exception, the host *E. coli* strain used for protein expression was Tuner(DE3)^TM^ and not Origami(DE3)^TM^ (Invitrogen, Carlsbad CA). The vaccine met the purity criteria for human-use injectables. Residual endotoxin content of the vaccine was 0.004 EU/µg protein by gel clot assay and purity of the protein was >95% by SDS-PAGE. The protein solution was transported from WRAIR, Silver Spring MD to CDC Atlanta GA, on dry-ice. Figure 1 shows the high level purity of the vaccine protein by SDS-PAGE under non-reducing and reducing conditions, and evidence of correct folding is shown by positive reactivity of the non-reduced protein to a conformation dependent monoclonal antibody 4G2dc1[21].

### Vaccination

Recombinant 3D7 AMA-1 protein was formulated with either Montanide ISA720 (ISA) (Seppic Inc, Paris) or AS02_A_ (GlaxoSmithKline Biologicals, Rixensart, Belgium) Adjuvant System. For ISA720 formulation the antigen in PBS was mixed with the adjuvant in the ratio of 30∶70 v/v and vortexed for 10 min to form a thick emulsion. For the AS02_A_ formulation, the lyophilized antigen was directly suspended in 0.5 ml of the adjuvant. The control vaccine consisted of PBS formulated with ISA720. Three doses of the vaccine, each containing 40 µg of AMA1 were administered intra-muscular on days 0, 28 and day 84, 0.25 ml vaccine on each thigh. Immunization sites were monitored daily for adverse local reaction and the weight of the animals was recorded at 4 wk interval during the study. Animals were bled for preparation of serum, complete blood counts and on defined occasions for clinical laboratory values, every two weeks beginning four weeks before the first immunization and continuing until the study was completed.

### Challenge and Determination of Parasitemia

Three groups of six monkeys were challenged with FCH/4 strain parasites; the groups designated as PBS+ISA_FCH/4_, AMA+ISA_FCH/4_ and AMA+AS02_A-FCH/4_. Two other groups were challenged with FVO strain parasites; these were designated as PBS+ISA_FVO_ and AMA+ISA_FVO_. Ring-stage parasitized erythrocytes from a donor monkey were diluted in sterile RPMI 1640 tissue culture medium. On day 112 of the study 50,000 and 10,000 ring stage parasites of the FCH/4 and FVO strain, respectively, were administered intravenously into the monkeys via injection into the femoral vein. Beginning 3 days after the intravenous challenge, Giemsa-stained blood smears were made to establish the number of parasites per µL blood by the quantitative thick film or by thin film, when parasite counts exceeded ∼80,000/ µL [19]. Daily blood smears were prepared and evaluated for 56 days after the FVO and FCH/4 challenge. Monkeys that developed high-density parasitemia (>200,000 parasites/µL of blood) were treated with mefloquine (Roche Laboratories, Nutley, N.J.) and quinine (Marion Merrel Dow, Inc., Kansas City, Kans.). Animals that developed anemia were cured with drugs and treated by iron supplementation and transfusion of whole blood if the hematocrit fell below 20%. Iron supplementation is a standard practice by veterinary staff for the treatment of malaria-induced anemia.

### ELISA

Plates coated with either 3D7 or FVO AMA-1 (50 ng/well) were blocked with 5% BSA-PBS for 1 hr. *Aotus* sera were serially diluted four-fold starting from 1∶1000 dilution and 75 µl of the dilution was incubated in the wells for 2 hrs at room temperature. The plates were washed thrice with PBS+0.05% Tween-20. Anti-*A. nancymaae* IgG heavy-plus-light chain peroxidase-labeled conjugate was diluted 1∶10,000 in PBS-Tween and 50 µl/well was incubated for 1 h at room temperature. Plates were washed and developed by the addition of 50 µl of 2,2′-azino-bis3-ethyl benzthiazoline-6-sulfonic acid (ABTS) peroxidase substrate system (KPL, Gaithersburg, MD). The optical density at 415 nm (OD_415_) was measured after 30 min using a microplate reader (Molecular Dynamics, Sunnyvale CA). The antibody endpoint titer was calculated as the serum dilution that produced an OD_415_ of 0.5 absorbance units in the ELISA assay using a 4 parameter curve fitting model in the Softmax software (Molecular Dynamics). The 0.5 OD_405_ fell in the linear part of the dilution curve.

### Expression of Chimeric Proteins and Domain ELISA

AMA1 consists of 3 disulphide bonded domains, D1, D3 and D3 [22]. In order to map the immune response to the various domains of AMA1, we produced 5 chimeric proteins. These chimeric proteins display the individual *P. falciparum* 3D7 AMA1 D1, D2, D3, D1+2 and D2+3 on a *P. berghei* scaffold. The design, expression and purification of the chimeric proteins will be described elsewhere. The domain ELISA utilized the chimeric AMA1 proteins as coat antigens. Various chimeras at 50 ng/well were coated on the ELISA plates and the assay was done as above.

### IFA

IFA slides were prepared from FVO and 3D7 asexual parasites grown to mature schizonts in vitro by depositing the infected erythrocytes into the wells and drawing the excess solution leaving a thin layer of cells that were allowed to air dry. Prepared slides were stored at −80°C until thawed for use in the IFA analysis. Sera were diluted 1∶32 with 0.01M PBS, pH 7.2, and two fold dilutions continuing to 1∶65,536 were placed in the wells for 1 hour in a moist chamber. Slides were washed in PBS and a 1∶100 dilution of FITC-labeled goat anti-Aotus monkey IgG was incubated in each well for another 1 hour in a moist chamber in the dark. After a second washing cycle the slides were dried, anti-fade mounting medium was applied and cover slip was mounted. Slides were then examined under a UV microscope. The dilution in the last well showing distinct fluorescence was taken as the IFA titer.

### GIA

Sera were thawed at 37°C in a water bath and heat inactivated for 20 minutes at 56°C. Ring stages at 0.2% parasitemia and normal RBC at 2% hematocrit were seeded in a 96 well plate with a final culture volume of 40 µl. Sera was tested at a final concentration of 10% against the 3D7 strain of *P. falciparum* and 5% against the FVO strain in a one cycle GIA (FVO was inhibited by normal Aotus sera above 5% v/v). The plate was sealed in a gas filled plastic bag and incubated under static conditions for 40 hrs [23]. Ring stages of the parasite were stained by adding 5 µl of the culture to 500 µl of 0.25X SYBR green dye in pH 7.4 PBS containing 10 mM EDTA and 10 mM glucose [23], [24]. Following 45 minute incubation in a shaking incubator maintained at 37°C, parasites were quantified by flow-cytometry (488/530 nm) using a BD FACScalibur (Franklin Lakes, NJ) gated on forward scatter for 40,000 RBC. Percent inhibition of invasion was calculated as, [1-(percent ring stage parasitemia in test well/percent ring stage parasitemia in pre-immune serum containing control well)].

### Statistical Analysis

Data for the three FCH/4 challenged groups was evaluated by analysis of variance to discern if differences existed among the various groupings of data; multiple group comparisons were then made by Tukey's posttest. Data of the 2 FVO challenged groups were compared by Mann–Whitney U test. Correlation between Log ELISA and cumulative day 11 parasitemia were established using the Spearman rank correlation. Plots were produced using Prism Graphpad (La Jolla, CA) or Microsoft Excel software (Redmond, WA).

## Results

### Sequence Differences between the Vaccine and Challenge Strain AMA1 Proteins

**Figure 2:**
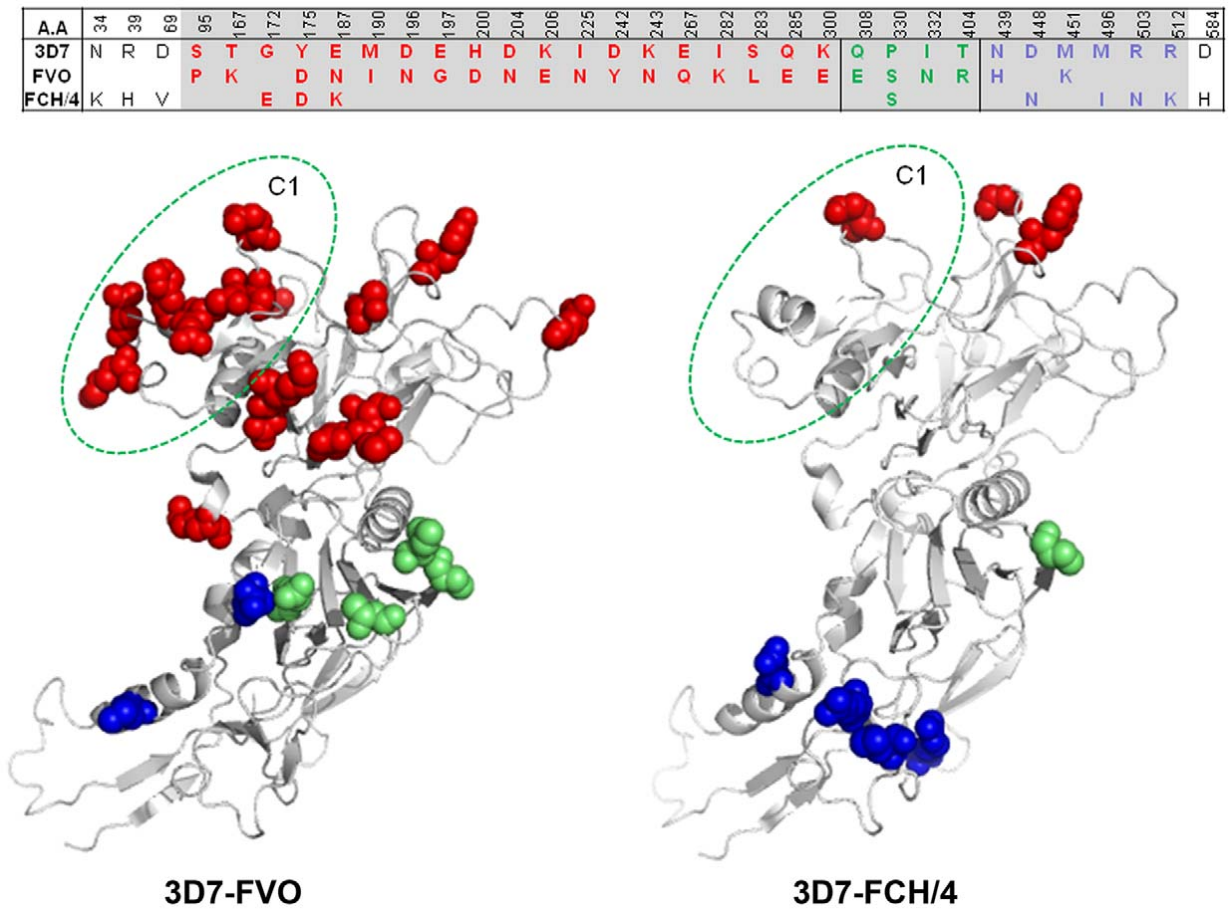
The FCH/4 strain AMA1 is more homologous to 3D7 AMA1 as compared to FVO strain AMA1. Top panel shows the amino acid differences between 3D7, FVO and FCH/4 AMA1. Polymorphisms within the grey cells are included in the sequence boundary of 3D7 AMA1 vaccine, amino acid 83–531. The disulphide bonded domains (D1, D2 and D3) are marked by thick lines. D1 (amino acid 95–300), D2 (308–404) and D3 (439–584). Lower panel shows the crystal structure of AMA1 (amino acids 97–531) with the location of the 3D7-FVO (left) and 3D7-FCH/4 (right) amino acid differences shown as solid balls (D1 polymorphisms - red; D2 - green and D3 polymorphisms - blue). The residues of the C1 cluster (187, 190, 196, 197, 200, 204, 206 and 225) are circled in green.

It was necessary to examine the potential protective effects of the human 3D7 vaccine against heterologous strains *of P. falciparum*, FCH/4 and FVO, because *P. falciparum* 3D7 parasite is not adapted to grow in Aotus monkeys. There are 12 amino acid differences between FCH/4 and 3D7 AMA1 as compared to 24 amino acid differences between FVO & 3D7 AMA1 (Figure 2, top panel). Since the 3D7 AMA1 vaccine consisted of amino acids 83–531 (grey cells in Figure 2), the sequence differences between the vaccine and the target strain within this sequence boundary were mapped to the crystal structure of AMA1 [25], [26] (Figure 2, bottom panel). Eighteen of the 24 3D7-FVO amino acid differences localized to domain-1 as compared to 3 out of the 12 3D7-FCH/4 differences. Hence, the 3D7 and FCH/4 domain-1 and domain-2 sequences are very similar. Within domain-1 the critical antigenic escape residues of AMA1 map to a group of polymorphisms within a three dimensional cluster termed C1, shown in Figure 2 as circled residues [27]. One of the 3D7-FCH/4 differences (residue 187) mapped to C1 as compared to 8 of the 3D7-FVO differences (residue numbers 187, 190, 196, 197, 200, 204, 206, 225). It was anticipated that the 3D7 vaccine was more likely to be effective against the C1-similar FCH/4 strain.

### Reactogenicity and Immunogenicity

The 3D7 AMA1 vaccine formulated with either ISA or AS02_A_ adjuvants was well tolerated by the non-human primates, with no indication of vaccine induced weight loss and only minor local reactions, primarily with ISA. Two of the monkeys died during the course of vaccination due to causes unrelated to the vaccine antigen; both deaths occurred in the PBS+ISA_FVO_ control group. Monkey AI-3190 died suddenly 10 days post the first immunization with PBS+ISA. Necropsy and histology revealed no or minimal pathology with the exception of a mild chronic interstitial nephritis. Monkey AI-3205 died 3 days prior to challenge after three immunizations with PBS+ISA. Histology indicated a severe sclerosing glomerulonephritis and the animal was under treatment with Lasix. These deaths were judged to be unrelated to the immunizations; acute and chronic nephritis are common in *Aotus* monkeys [28].

#### ELISA

**Figure 3:**
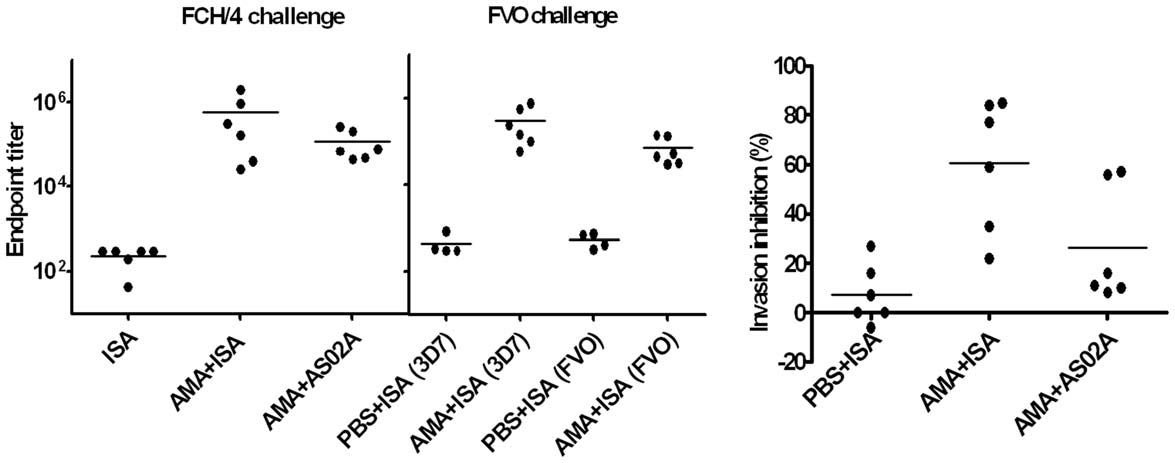
AMA1+ISA vaccine formulation induced high titer antibodies that inhibited homologous parasite invasion. Left panel shows ELISA endpoint titer of day-of-challenge sera and the group-mean (line). In the FCH/4 challenge group ELISA was done using 3D7 AMA1 coat antigen and for the FVO strain challenge group, ELISA was done using 3D7 and FVO AMA1 coat antigens (shown in parentheses on the *x-axis*). Both ELISA panels share the same *y axis* scale. Right panel shows the GIA activity of the day-of-challenge sera of the FCH/4 challenged groups measured at 10% serum concentration against 3D7 strain parasites.

Sera collected on the day-of-challenge were assayed using 3D7 AMA1 coated plates. The AMA+ISA_FCH/4_ titers ranged between 26,000–2,000,000 (median = 236,178) and the AMA+AS02_A-FCH/4_ group titers ranged between 45,000–260,000 (median = 73,773). Although the mean titer of AMA+ISA_FCH/4_ group (577,456) was 5 times higher than AMA+AS02_A-FCH/4_ group (116,423), this difference was not statistically significant (Figure 3, left panel and Table 1). The mean titer of the FVO challenged AMA+ISA_FVO_ group was 303,034 on 3D7 AMA1 coated plates and significantly lower, 69,696, against the heterologous FVO AMA1 plate antigen (Mann Whitney U test p = 0.02) (Figure 3, middle panel and Table 1).

**Table 1:** Immunogenicity and efficacy data of individual animals.

Group	Animal	ELISA (X1000)		IFA		GIA		Parasites/µL (day 4–11)		
		3D7	FVO	3D7	FVO	3D7	FVO	Mean	Cumulative	Peak
PBS+ISA FCH/4	AI-3162	0.3	0.3	64	64	7%	nd	52016	416130	280000
	AI-3166	0.3	0.5	128	64	16%	nd	23996	191970	59580
	AI-3168	0	0.3	<32	32	27%	nd	68358	546860	296000
	AI-3178	0.1	0.5	32	<32	0%	nd	11468	91740	42300
	AI-3200	0.3	0.3	32	32	0%	nd	28413	227300	116000
	AI-3210	0.3	0.2	<32	<32	−6%	nd	55668	445350	160000
AMA+AS02_A_ FCH/4	AI-3057	70	36	4096	512	16%	nd	37246	297970	164000
	AI-3172	78	17	8192	256	10%	nd	12860	102880	72000
	AI-3177	261	133	8192	2048	57%	nd	789	6310	4320
	AI-3063	197	83	8192	4096	56%	nd	9558	76460	44000
	AI-3195	45	25	2048	256	11%	nd	7485	59880	15840
	AI-3211	48	30	1024	128	8%	nd	1461	11700	7650
AMA+ISA FCH/4	AI-3176	2015	481	32768	8192	85%	nd	0	0	0
	AI-3179	912	352	16384	4096	84%	nd	4	30	10
	AI-3181	309	144	65536	4096	77%	nd	30	240	240
	AI-3182	163	116	8192	4096	59%	nd	20498	163980	105000
	AI-3191	40	14	8192	128	22%	nd	43531	348250	140000
	AI-3226	26	18	4096	1024	35%	nd	26935	215490	84000
PBS+ISA FVO**	AI-3054	0.3	0.7	64	64	13%	4%	107451	859610	640000
	AI-3163	0.8	0.7	256	256	−11%	0%	11843	94740	87000
	AI-3185	0.3	0.4	128	128	13%	5%	60233	481860	288000
	AI-3199	0.3	0.3	32	32	−2%	−1%	94416	755330	520000
AMA+ISA FVO	AI-3055	733	129	16384	2048	63%	4%	162510	1300080	1040000
	AI-3173	140	44	8192	2048	24%	−2%	38165	228990	208000
	AI-3174	557	134	8192	1024	67%	15%	17030	136240	56000
	AI-3193	232	52	8192	2048	48%	2%	54651	437210	320000
	AI-3203	57	29	8192	4096	6%	0%	63575	508600	400000
	AI-3209	98	31	2048	2048	8%	3%	55638	445110	348000

Vaccine and challenge groups, animal ID, ELISA endpoint titer against 3D7 or FVO AMA1 coated plates, IFA titer against 3D7 and FVO schizonts, GIA activity against *P. falciparum* 3D7 or FVO target parasite and parasite burden (mean, peak and cumulative counts) between days 4 and 11 post challenge are shown. ** Two animals in the PBS+ISA_FVO_ group died due to unrelated causes during the vaccination phase. GIA was not done (nd). The ELISA titer values are 1000X.

#### IFA

Immuno-fluorescence titers of the day-of-challenge sera are shown in Table 1. The AMA+ISA_FCH/4_ group IFA titers ranged between 4,096–65,536 (median = 12,288). The AMA+AS02_A-FCH/4_ group titers ranged between 1,024–8,192 (median = 6,144). Although the mean IFA titer of AMA+ISA_FCH/4_ group (22,528) was 4 times higher than that of the AMA+AS02_A-FCH/4_ group (5,291) this difference also did not reach statistical significance as was observed with ELISA titers. The mean IFA titer of the FVO challenged AMA+ISA_FVO_ group was 8,533 against 3D7 schizonts and significantly higher than the 2,219 titer against the heterologous FVO schizonts (Mann-Whitney U test p = 0.01).

#### GIA

The day-of-challenge sera of the FCH/4 challenged groups were analyzed for their ability to inhibit invasion of 3D7 strain merozoites using growth inhibition assay (GIA) (Figure 3, right panel and Table 1). The mean GIA activity of AMA+ISA_FCH/4_ group was higher than both the control PBS+ISA_FCH/4_ and the AMA+AS02_A-FCH/4_ group (ANOVA followed by Tukey's multiple comparison test, p<0.05). The mean GIA activity of AMA+AS02_A-FCH/4_ group sera was indistinguishable from the PBS control group. The sera from the AMA+ISA_FVO_ group had GIA activity similar to the AMA+ISA_FCH/4_ group however the GIA activity against the heterologous FVO target parasite was not detectable (Table 1).

Overall these results indicated that the 3D7 AMA1 vaccine was inducing an efficient antibody response in Aotus monkeys, with higher levels of antibody detected with the ISA adjuvant, which resulted in efficient inhibition of the homologous 3D7 parasites. Levels of cross-reacting antibodies against FVO AMA1 protein were ∼5x lower, which was insufficient for *in vitro* growth inhibition of the FVO parasite.

### Analysis of Parasitemia Profiles

In order to determine if the 3D7 AMA1 vaccine had induced protection against FCH/4 and FVO parasite challenge, parasitemia profiles were examined for individual animals within the different immunization groups.

#### PBS+ISA_FCH/4_ group

**Figure 4:**
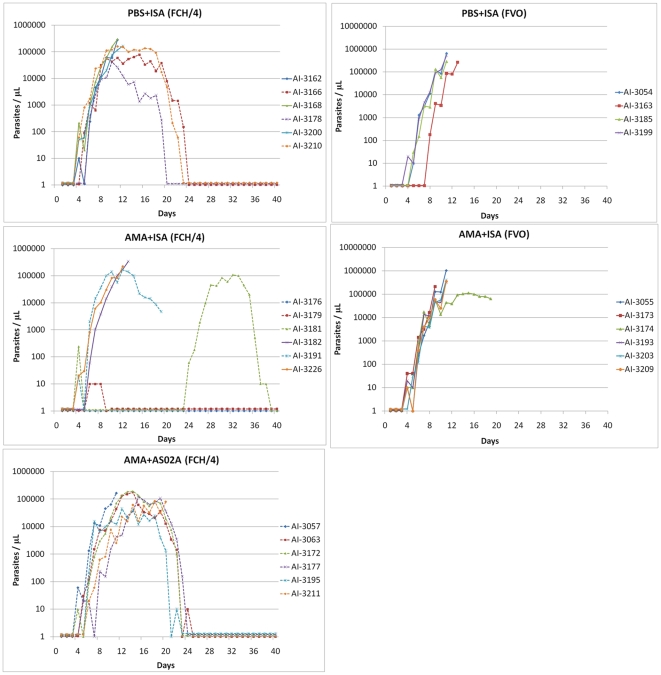
Parasitemia profiles of FCH/4 and FVO challenged groups. Daily parasitemia of animals plotted against days-post-challenge (days). Solid lines represent a virulent parasitemia profile that required drug treatment for high parasitemia (>200,000/µL) within 15 days of challenge. Broken lines represent a slower progressing and self-limiting infection profile. The challenge strains are indicated in parentheses (FCH/4 or FVO). Two monkeys in the AMA+ISA_FCH/4_ group (AI3181, AI-3176) were re-challenged on day 16.

Inoculation of 50,000 *P. falciparum* FCH/4 strain parasitized RBC in the control PBS+ISA_FCH/4_ group led to detectable parasitemia in all monkeys by day 5 post challenge. During the following days two different parasitemia profiles were observed. Three monkeys (AI-3162, 3168, 3200; solid lines in Figure 4) developed a rapidly rising ‘virulent’ parasitemia that required drug treatment within 15 days of challenge. The peak parasitemia of AI-3162, AI-3168, AI-3200 were 280,000, 296,000 and 164,000 respectively. Three remaining monkeys (AI-3166, 3178, 3210; broken lines) had a rapidly growing but ‘self-limiting’ parasitemia profile (defined as not requiring drug treatment for high parasitemia), and eventually becoming subpatent by day 24, 20, and 23, respectively. AI-3166 and AI-3210 had brief recrudescent infections by days 50 and 43. Peak parasitemia in AI-3166, AI-3178 and AI-3210 were 78,300, 42,300 and 160,000 parasites per µL. The mean peak parasitemia of the 3 animals with virulent profile was 246,667/µL, and that of the 3 animals with a self-limiting profile was 93,533/µL.

#### AMA+ISA_FCH/4_ group

Monkeys AI-3182 and AI-3226 had the virulent parasitemia profile that required treatment on day 12 and 13 and thus were classified as not protected. Monkey AI-3191 had a self-limiting parasitemia profile reaching 168,000 parasites per µL that was given anti-malarial treatment on day 19 due to severe anemia and, therefore, was also not considered protected. Monkey AI-3176, remained slide negative after the first challenge and was then re-challenged with additional 50,000 parasitized erythrocytes on day 16. AI-3176 remained smear-negative throughout the trial showing sterile immunity. Monkey AI-3179 also showed protection, it had a short period of low level parasitemia (∼10 parasites/µL for 3 days) that self cured by day 8, following which this animal remained subpatent. Monkey AI-3181 was patent (240 parasites/µL) only on day 4 and then remained smear-negative. AI-3181 was also re-challenged on day 16 with 50,000 FCH/4 parasites, following which a parasitemic phase was observed starting at day 24, peaking at 108,000 parasites per µL on day 32, and then self-resolving by day 38. This monkey was protected against the first challenge and as indicated by its sub-patency and also against the re-challenge as indicated by a delay in patency (7 days *vs.* 3–4 days).

#### AMA+AS02_A-FCH/4_ group

All animals of the AMA+AS02_A-FCH/4_ group became smear positive between day 4 and day 6. Although no sterile protection was observed, the parasitemia profiles indicated that all 6 monkeys mirrored the self-limiting parasitemia profile (not requiring drug treatment for high parasitemia >200,000/µL) as compared to 3 of 6 in the control group (Figure 4). Peak parasitemia ranged from 44,600 to 192,000 per µL. Monkeys AI-3057 and AI-3211, despite a self-limiting parasitemia profile received drug treatment on day 11 and day 20, respectively, due to severe anemia. Thus, this group perhaps showed a degree of partial protection. However the mean peak parasitemia of the six self-limiting parasitemia profile monkeys in the AMA+AS02_A-FCH/4_ group (130,773/µL), was not different from the 3 control group animals (93,533/µL) that had a self-limiting profile (2 tailed t-test, p = 0.6). *PBS+ISA_FVO_ group:* There were only 4 animals in the control PBS+ISA_FVO_ group at the time of challenge due to the death of 2 monkeys during the vaccination phase. Infection induced by inoculating 10,000 FVO strain parasitized RBC led to a rapidly rising virulent parasitemia profile in all 4 control monkeys with anti-malarial drug cure given between day 11 and 13 (Figure 4).

#### AMA+ISA_FVO_ group

All of the 6 animals had a virulent parasitemia profile comparable to the PBS+ISA_FVO_ control group. Five monkeys had to be treated for high parasitemia on day 9 or 11. One other monkey (AI-3174) peaked at 112,000 parasites/µL and on day 19 was treated with MQ and quinine for infection and severe anemia. No protection was observed against heterologous FVO strain challenge (Figure 4).

### Parasite Burden and Correlates of Protection

**Figure 5:**
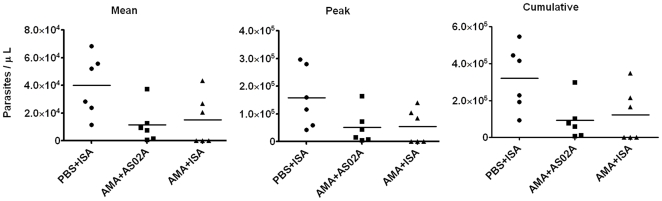
3D7 AMA1 vaccination reduced the parasite burden of the FCH/4 strain. Mean, peak and cumulative parasitemia (parasites/µL) of individual animals and their mean (line) between days 4–11 post challenge.

Mean, peak and cumulative parasite levels were determined for group-wise comparisons. The first monkey in the FCH/4 challenged groups became parasitemic on day 4 and was drug treated on day 11, hence the relative parasite burden of the FCH/4 challenged groups was compared between days 4–11 (Figure 5 and Table 1). There was a trend towards lower mean, peak and cumulative parasitemia in the AMA+ISA and AMA+AS02_A_ groups compared to the controls. However this difference in parasite burden did not reach statistical significance (p values for ANOVA for repeated measures design for mean, peak and cumulative parasitemia was 0.06, 0.08 and 0.06 respectively). For the FVO challenge groups the mean, peak and cumulative parasitemia between days 4–11 of the AMA+ISA_FVO_ and the PBS+ISA_FVO_ groups were compared by Mann-Whitney U test (not plotted). No difference in the parasite burden was observed between the vaccine and the control group following FVO parasite challenge.

**Figure 6:**
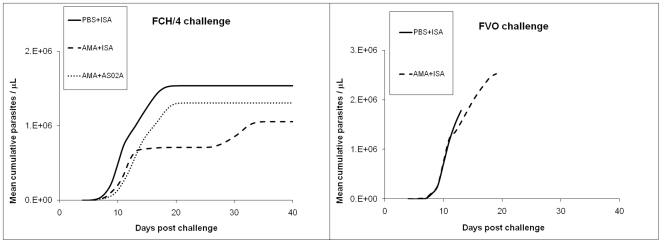
3D7 AMA1 vaccination slowed growth rate of FCH/4 but not FVO strain. Group-wise mean cumulative parasitemia of the FCH/4 (left panel) and FVO (right) challenged animals plotted against days post challenge. Solid line, PBS control group; broken line, AMA+ISA; dotted line, AMA+AS02_A_.

A plot of the mean cumulative parasitemia revealed further differences between the FCH/4 challenged groups (Figure 6, left panel.) The rate of parasite growth indicated by the steepness of the cumulative parasitemia curve indicated that AMA1 vaccine had slowed the rate of FCH/4 multiplication in both the adjuvant groups as compared to the PBS control vaccine. The mean cumulative parasitemia on day 11, for the PBS-ISA_FCH/4_ group (319,892) was higher than the AMA+ISA_FCH/4_ group (121,332; Mann-Whitney U test p = 0.06), and the AMA+AS02_A-FCH/4_ group (92,533, p = 0.02). Among the 2 FVO strain challenged groups, there was no difference in the growth rates between the AMA1-vaccinated and the control group (Figure 6, right panel).

**Figure 7:**
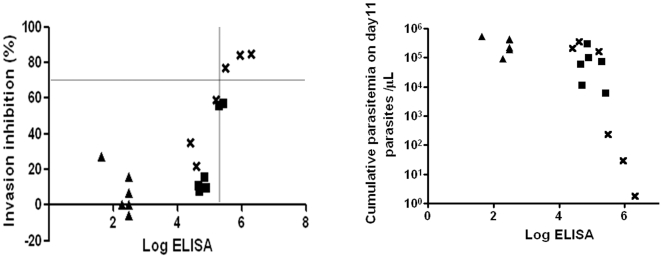
ELISA titers positively correlate GIA activity and negatively correlate parasitemia. Left panel shows GIA activity of the FCH/4 challenged animals plotted against log ELISA titer. Group symbols: PBS+ISA (triangle), AMA+ISA (cross) and AMA+AS02_A_ (square). The three monkeys that were protected in the study had GIA activity of >70% and ELISA titer of >300,000, cut-off indicated by grey lines. Right panel shows the correlation between log ELISA endpoint titer and the cumulative day 11 parasitemia for the 18 FCH/4 challenged animals.

In this study, 18 animals in 3 groups were challenged with FCH/4 strain. Three monkeys in the AMA+ISA_FCH/4_ group were protected: AI-3176 and AI-3179 had greatly reduced or no parasite burden and AI-3181 had a delayed parasitemic phase. The GIA activity of serum from these 18 monkeys was plotted against the log ELISA titer (Figure 7, left panel). Sera from these three monkeys had >70% GIA activity, >300,000 ELISA titer and IFA titer >8,192 (Table 1). There was also a negative correlation between the cumulative parasitemia on day 11 and the log ELISA endpoint titer (Figure 7, right panel). Spearman rank test confirmed the statistical significance of this correlation (rho factor r = −0.780 and p = 0.000134).

### Domain-Wise Mapping of Antibody Responses

**Figure 8:**
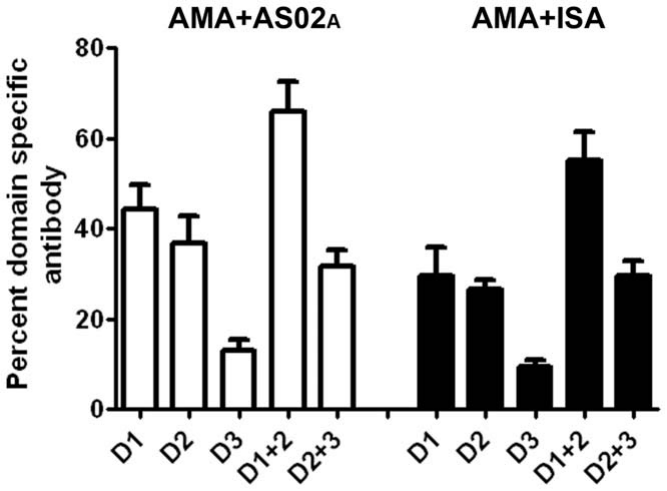
Domain-specificities of AS02_A_ and ISA720 induced anti-AMA1 were similar. End-point titer was determined against chimeric proteins displaying *P. falciparum* domains 1, 2, 3, 1+2 or 2+3 on *P. berghei* AMA1 scaffold (D1, D2, D3, D1+2, D2+3 chimeras respectively). Domain-specific titer was calculated by expressing the domain-specific end-point titer as a percentage of titer against the full-length 3D7 AMA1 protein. Mean and standard error for 6 animals per group was plotted.

To determine the differences in specificity of antibodies induced by the two adjuvants, and to explore if a particular domain-specific pattern correlated with protection, chimeric AMA1 proteins were used to determine the ELISA end-point titer against 3D7 AMA1 domains-1, 2, 3, 1+2 and 2+3 (termed D1, D2, D3, D1+2, D2+3 proteins respectively). The mean end-point titer of 12 AMA1 vaccinated monkeys against the protein chimeras D1, D2, D3, D1+2 and D2+3 were 45053, 48971, 14708, 103803 and 56107 respectively. Although, the contribution of antibodies that cross-react with the *P. berghei* AMA1 in this mapping analysis cannot be ruled out, the mean endpoint titer of the 12 monkeys against the recombinant scaffold protein *P.berghei* AMA1 was significantly lower, 3202, than the chimeric proteins that display PfAMA1 epitopes. To normalize the individual animal titers, the domain-specific end-point titer was expressed as a percentage of the titer against the full-length AMA1 protein. The mean domain-specific titer of AMA+AS02_A-FCH/4_ and AMA+ISA_FCH/4_ groups was plotted in Figure 8. Analysis of variance, followed by Tukey's posttest showed no difference in domain specificity of antibodies induced by AS02_A_ *vs.* ISA70 adjuvant. Among the single domains, the reactivity pattern was D1 = D2>D3 and among the double domains, reactivity pattern was D1+2>D2+3. Proportion of antibodies against the D1+2 was highest between the domains and was statistically indistinguishable from the titer against the full-length antigen for both AMA+ISA and AMA+AS02_A_ groups (Tukeys multiple comparison, p>0.05), indicating that the majority of the antibodies that bind to the full-length antigen recognize epitopes formed by residues present on domains 1 and 2.

**Figure 9:**
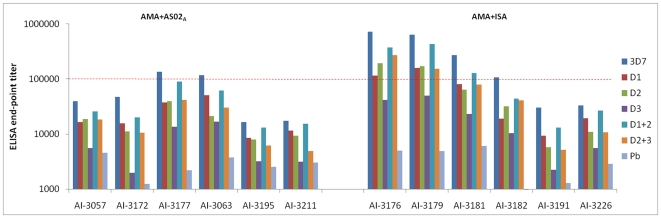
Domain1+2 end-point titer of protected animals exceeded 100,000. FCH/4 challenged AMA+AS02_A_ and AMA+ISA group titers against full-length 3D7 AMA1 (3D7) or chimeras D1, D2, D3, D1+2, D2+3 that display the corresponding *P. falciparum* 3D7 strain AMA1 domains on a *P. berghei* AMA1 scaffold (Pb). End-point titers against the *P. berghei* AMA1 scaffold protein are also plotted. Animals protected in the AMA+ISA group (AI-3176, AI-3179 and AI-3181) had the highest D1+2 end-point titer (red dotted line).

Figure 9 shows the end-point titers of all 12 AMA1 vaccinated and FCH/4 challenged monkeys. Individual serum titers showed that protection did not correlate with a unique domain specific antibody induction pattern, instead the titers in the AMA+AS02_A_ group were generally lower against all domains and the protected monkeys (AI-3176, AI-3179 and AI-3181) had D1+2 titers exceeding 100,000 (Figure 9), suggesting that magnitude of antibody response to the full-length AMA1 and domains1+2 are a critical correlate of AMA1 based protection.

## Discussion

The 3D7 strain of *Plasmodium falciparum* grows vigorously *in vitro* and was used for the first genome sequencing of a malaria parasite [29]. This strain of *P. falciparum* also produces gametocytes and its sensitivity to chloroquine has made it an excellent choice as a challenge strain for human Phase 2a vaccine trials [30]. Despite numerous attempts made at the CDC Atlanta by Dr. William Collins and others, the 3D7 parasite could not be adapted to grow in the New World monkey models (personal communication). Hence, in order to test the efficacy of a 3D7 strain based AMA1 vaccine, we utilized two heterologous strains FCH/4 and FVO.

The FVO strain is highly virulent in *Aotus nancymaae*, whereas, the FCH/4 strain is less well adapted. In this study, a dose of 50,000 FCH/4 strain parasites produced virulent infection requiring drug treatment in 3 of 6 animals in the control group, while the remaining 3 animals self-cured after developing high parasitemia (Figure 4). In comparison, only 10,000 parasites of the FVO strain were sufficient to produce a rapidly rising virulent infection in 4 of 4 control monkeys, all of which required drug treatment (Figure 4). Positive efficacy of the AMA+ISA vaccine against a FCH/4 strain challenge was observed where protection, as measured by a reduced parasite burden, was 50% and as measured by sterile protection, was 16% (Figure 4, 5 and 6). The animals in the AMA+AS02_A_ group had a reduced rate of parasitemia increase that did eventually reach a high mean peak parasitemia that was surprisingly higher than the parasitemia of the control group monkeys with a self-limiting profile. None of the animals in the AMA+AS02_A_ group needed treatment for the hyper-parasitemia, although two were treated for severe anemia. ELISA and IFA titers showed that anti-3D7 AMA1 antibodies had lower recognition of the FVO strain AMA1, and, this was associated with 0% efficacy of the AMA+ISA vaccine against the more virulent FVO strain.

Although FCH/4 is less virulent than the FVO strain, it was chosen as a one of the two challenge strains because it was the nearest in amino acid sequence of AMA1 to 3D7 among a group of monkey adapted *P. falciparum* strains available for challenge infections. The structural distribution of the amino acid differences between 3D7 and FCH/4 *versus* 3D7 and FVO was analyzed to rationalize the observed pattern of heterologous protection. We have previously shown that the antigenic escape residues of 3D7 AMA1 reside within a small three dimensional cluster called C1 [27] (Figure 2). This cluster contains the most polymorphic sites (residues 187, 190, 196, 197, 200, 204, 206, 225) of AMA1. Interestingly, only one amino acid difference between 3D7-FCH/4 mapped to the C1 cluster (Figure 2), as compared to 8 differences between 3D7-FVO. Indeed, the majority of the differences between 3D7 and FCH/4 in AMA1 mapped to domain-3 (residues 448, 496, 503, 512). Published cross-strain inhibition data using anti-3D7 antibodies against *P. falciparum* D10 strain target parasites has shown that three out of the four 3D7-FCH/4 domain-3 amino acid differences confer no escape advantage to the D10 strain parasite in a cross-strain GIA against anti-3D7 AMA1 [31]. The FCH/4 strain was, therefore, an appropriate choice to study the protective efficacy of 3D7 strain AMA1 vaccine formulation.

The most likely cause of vaccine failure in the FVO challenged animals was therefore antigenic escape from strain-specific 3D7 AMA1 antibodies. Alternatively, there may be differences in the relative susceptibility of FCH/4 and FVO strains to invasion inhibitory AMA1 antibodies. We have shown previously that affinity purified antibody required for 50% invasion inhibition was lower for the 3D7 strain parasite, as compared to the FVO strain [27]. This suggested that FVO parasite may be inherently more resistant to inhibition by anti-AMA1. Most importantly future AMA1 vaccines need to be evaluated for their ability to inhibit multiple strains of the parasite as well as some primary isolates that may use multiple invasion pathways.

Three out of the 12 AMA1 vaccinated monkeys (compared to 0 of 6 adjuvant control monkeys) were strongly protected against a relatively homologous, yet weak, challenge strain FCH/4. The protected monkeys had the highest GIA activity, high IFA titer and highest ELISA titer against full-length and domains1+2 of AMA1. There was a negative correlation between mean parasite count and ELISA titer and protection correlated with a threshold antibody titer, which was achieved in some monkeys of the ISA720 group, but not in the AS02_A_ group. An AMA+AS02_A_ formulation failed to induce sterilizing immunity in humans in a Phase 1/2a trial [16], although it reduced the parasite growth rate, as was observed for AS02_A_ formulation group in this Aotus study. It is difficult to compare the absolute antibody titers between species, but GIA activity of the sera can be more accurately compared. In the Phase1/2a trial, the mean GIA activity measured using 20% v/v serum concentration against the 3D7 strain parasite was 30% (range 10–40%) [16], which is concordant with a dose escalation Phase 1 trial where the AMA+AS02_A_ vaccine induced GIA activity in the 30–40% range, also at 20% serum concentration [14]. In a recently published human Phase 1 trial with FVO AMA1 vaccine, adjuvanted with IS720 and AS02, the mean GIA activity was in the 20–30% range at 10 mg/ml IgG [32], for both adjuvant groups. The 10 mg/ml concentration is close to the physiological concentration of IgG in the serum, and our data suggests that significantly higher level of invasion inhibition (>70% activity at one tenth the equivalent serum concentration, 10% v/v) is required to confer protection. The sera from the Phase1/2a trial [16] also failed to cause cross-strain inhibition of the FVO parasite, which was also observed in our non-human primate study reported here.

Viral neutralization titer is generally defined as the serum dilution that neutralizes 50% of virus. It is easier and more accurate to titer out antibodies to a 50% end-point than to measure higher levels of neutralization or inhibition directly. Harmonization of assay protocols for several virus diseases has allowed comparison of vaccine efficacy between trials. A 50% plaque reduction neutralization titer of >120 (1∶120 serum dilution) correlates with protection against measles [33] and a 50% neutralization titer of >33 against the influenza virus also correlates protection [34]. Our data suggests that 70% inhibition (neutralization) at 1∶10 serum dilution is adequate to protect against a malaria challenge in Aotus monkeys. Limitations in availability of Aotus sera did not allow us to use higher concentrations of serum antibodies. We expect that antibodies in undiluted sera would have greater than 90% inhibition of FCH/4 in GIA, although we cannot rule out other immune mechanisms. This result is not surprising because the growth-rate of *P. falciparum* parasites *in vitro* is between 6–15 fold per cycle, and a high level of GIA activity is needed to induce solid protection. Thus far no human-use vaccine formulation has met this GIA activity. If viral neutralization titers are an indication, malaria vaccine formulations need to generate significantly higher titers to induce sterile protection. This may be further complicated by other anti-malaria antibodies, prevalent in endemic populations, that block the anti-AMA1 mediated invasion inhibition [35]. Future Aotus trials using Montanide formulations of AMA1 can provide an opportunity to confirm the current findings and to establish a cut-off ELISA titer/GIA activity that human-use formulations must achieve, prior to entering advanced human trials.

AMA1 antibodies also need to be capable of inhibiting invasion across a broad range of parasite strains. A Phase 2b study using a bi-allelic 3D7+FVO AMA1 vaccine in 300 Malian children, showed no impact of vaccination on the frequency of parasitemic episodes. The 3D7 based AMA1 vaccine used in our manuscript has been evaluated in a series of clinical trials, culminating in a Phase 2b trial in Mali, Africa, an area of significant *P. falciparum* genetic diversity (Chris Plowe, University of Maryland, personal communication). If the current generation of 3D7 and FVO AMA1 based vaccines [36], fail to reduce parasite burden in children, structural vaccinology [27] and population biology [37] approaches will be needed to engineer a second generation antigen, that induces higher titer of cross-reactive inhibitory antibodies. Aotus monkey trials can serve as an important down selection-criteria to make critical go-no-go decisions, prior to testing the next generation of AMA1 based vaccines in humans. Novel formulation and delivery platform selection studies can benefit from a threshold GIA activity reported here, as a correlate of AMA1 based protection in the Aotus model.
